# An unusual complication of silver nitrate therapy for chyluria

**DOI:** 10.4103/0970-1591.32079

**Published:** 2007

**Authors:** Anant Kumar

**Affiliations:** Department of Urology and Renal Transplantation, Sanjay Gandhi Postgraduate Institute of Medical Sciences, Lucknow, India. E-mail: a_kumar99@yahoo.com

Sclerotherapy has a definite role in the management of chyluria. The commonly use agent in the past was silver nitrate which has also been successfully used for benign persistent haematuria. Any agent if not used properly will cause more damage than intended benefit. I have gone into details of all the complication reported by intrarenal silver nitrate instillation and realized that it was due to improper use.

Following were the most common mistakes.

1. Higher concentration- 3 and 5% of silver nitrate solution-should never be used.
2. Forceful instillation and injection of more than the pelvic volume leading to pyelovenous and pyelolymphatic reflux resulting into parenchymal and vascular damage.
3. Washing with normal saline and precipitating silver chloride, which is an opaque precipitate and causes obstruction.

We have done over 300-sliver nitrate instillation without any serious complication by adhering to basic rule;

1. Always use 0.5 or 1% silver nitrate solution
2. Never inject with pressure or more than 10 ml
3. Never wash with normal saline

We always passed a catheter and instilled three times a day for three days.

Only complication reported from SGPGI was a patient referred from elsewhere and had 3% instillation and washing by normal saline. For last few years we have been using Provodine Iodine and still using silver nitrate for resistant cases.
